# Breastfeeding self-efficacy as a dominant factor affecting maternal breastfeeding satisfaction

**DOI:** 10.1186/s12912-019-0359-6

**Published:** 2019-08-16

**Authors:** Siti Nurbayanti Awaliyah, Imami Nur Rachmawati, Hayuni Rahmah

**Affiliations:** 1 The Institute of Health Science Jenderal Achmad Yani, Cimahi, West Java 40633 Indonesia; 2 Master of Nursing Program, Faculty of Nursing, Universitas Indonesia, Jalan. Prof. Dr. Bahder Djohan. Kampus UI Depok, Depok, West Java 16424 Indonesia; 3 Department of Maternity and Women’s Health, Faculty of Nursing, Universitas Indonesia, Jalan. Prof. Dr. Bahder Djohan. Kampus UI Depok, Depok, West Java 16424 Indonesia

**Keywords:** Breastfeeding, Breastfeeding self-efficacy, Maternal breastfeeding satisfaction

## Abstract

**Background:**

One of the psychological benefits of breastfeeding for mothers and infants is to get the satisfaction of breastfeeding. Maternal breastfeeding satisfaction derives from the interaction and cooperation between mothers and their babies. This research aims to identify the maternal breastfeeding satisfaction and its influential factors.

**Methods:**

This study applied a cross-sectional design. Two hundred four breastfeeding mothers after four until 8 months postpartum were recruited using cluster sampling methods. Respondents completed the questionnaire of Maternal Breastfeeding Evaluation Scale (MBES) to identify maternal breastfeeding satisfaction. Factors affecting maternal breastfeeding satisfaction were identified using the following instruments: Breastfeeding Knowledge Questionnaire was used to identify knowledge on lactation, Infant Feeding Attitude Scale (IIFAS) to identify attitude toward lactation, and the Breastfeeding Self-Efficacy Scale-Short Form (BSES-SF) to identify breastfeeding self-efficacy.

**Result:**

We identified that 53.4% of the breastfeeding mothers had a high level of satisfaction. The results indicate that the household income, type of delivery, and breastfeeding self-efficacy was associated with breastfeeding satisfaction (*p* < 0.05). Breastfeeding self-efficacy is the most influential factors in maternal breastfeeding satisfaction (OR=16.64; CI 95% 7.65–35.94).

**Conclusion:**

Breastfeeding satisfaction is the satisfying feeling obtained during breastfeeding resulting from cooperation between the mother and the infant to fullfil desires or needs. Education and promotion of breast milk and breastfeeding provided by professional healthcare providers encouraged the successful of breastfeeding programs. Assistance by a health care provider or breastfeeding counselor during the seven contacts breastfeeding initiated during pregnancy until after delivery should be applied so that the mother is informed about breastfeeding. Breastfeeding satisfaction can keep the mother from continuing to breastfeed her baby for up to 2 years or more.

## Background

The World Health Assembly (2012) supported a resolution for comprehensive implementation of nutritional targets for mothers, infants, and children, as ratified in the Global Nutrition Targets 2025. Globally, only 38% of infants aged 0 to 6 months receive breast milk exclusively [1]. According to a report from the United Nations Children’s Fund (UNICEF), Indonesia, only 42% of infants in Indonesia of up to 6 months old are exclusively breastfed [2]. The average duration of exclusive breast milk nutrition is only 1 to 2 months, with the rate of breastfeeding decreasing after 2 months of age. The average duration of breastfeeding wordwide is 20.5 months, and the average duration of exclusive breastfeeding in Indonesia is more than 3 months [3].

Several factors contribute to the low rate of exclusive breastfeeding. The healthcare and social care professionals sometines assume that mixed feeding practice is more advantageous that exclusive breasfeeding. The other factors include the absence of support for breastfeeding in the workplace, the advertising of formula, and lack of knowledge of the women, partners, family members, healthcare providers and policy makers about the appropriate methods for, and the risks associated with, non-exclusive breastfeeding [1].

In reality, many women experience difficulties in breastfeeding. For the majority, it is not easy or natural [4]. A majority of women consider initial breastfeeding to be a painful, difficult and challenging experience [5]. Failure in breastfeeding is not only related to feelings of guilt [6] but also to the diminishing of maternal identity [7].

Failure in breastfeeding usually derives from problems faced by mothers and infants [8]. Breastfeeding problems may often occur up to 2 weeks (51%) and 6 weeks (49%) after birth and the perception of the lack of breast milk is the most common problem faced. Problems of breastfeeding are related to feelings of satisfaction in breastfeeding mothers, and these have an impact on early breastfeeding. Maternal breastfeeding satisfaction is defined as the perception by mothers of personal satisfaction and success in breastfeeding. Duration of breastfeeding determines the level of satisfaction for mothers who exclusively breastfeed for up to 2 months (84.6%) and 4 months (69.8%), even though the duration is lower than the recommended 6 months [9].

Some factors related to the duration are age, education, employment, household income, parity, and delivery type [10]. Moreover, knowledge, attitude, self-efficacy, intention and social support mothers receive also affect satisfaction [11]. Studies regarding breastfeeding satisfaction need to be carried out in Indonesia. Such research is crucial as satisfaction can provide benefits, including mental health benefits, for mothers and infants. Regarding mental and emotional wellbeing, it is the case that stimulation of the hormone oxytocin, which occurs in mothers and infants during breastfeeding, creates a pleasant experience for both, and motivates mothers to continue breastfeeding.

## Methods

This cross-sectional study aimed to determine the association between the dependent variable of breastfeeding satisfaction with independent variables of breastfeeding satisfaction factors consisting of age, education, employment, household income, parity, type of delivery, knowledge about breastfeeding, attitudes toward breastfeeding and breastfeeding self-efficacy of the mothers. A cluster sampling was applied in selecting 2478 breastfeeding mothers who spread over 62 Community Health Centers in Bandung, West Java, Indonesia. Primary sampling unit was two Community Health Centers, namely Pasir Kaliki and Babakan Sari. Secondary sampling units were breastfeeding mothers after 4 to 8 months of delivery. The inclusion criteria for the study were mothers who breastfeed their infant for 4–8 months after birth, marital status, and residence within a nuclear family. The exclusion criteria were mothers with physical problems such as bulbous nipples resulting from hypoplasia, or insufficient glandular. The other conditions, such as mothers who had a poor health condition and habitual smoking also were not included. This sampling method assigned 204 breastfeeding mothers from 34 Community Health Centers as respondents.

For measuring satisfaction was applied the Maternal Breastfeeding Evaluation Scale (MBFES) which developed by Leff, Jeffries, and Gagne (1994). MBES was designed to measure the positive and negative aspects of breastfeeding mothers to identify the importance of breastfeeding successful definition (Schlomer, Kemmere, & Twiss, 1999). This tool was translated into Bahasa Indonesia and tested for validity and reliability using Cronbach’s alpha, giving a value of 0.940. Low satisfaction if the value is less than 115, high satisfaction if the value is more than 116. The following was adapted questionnaires procured in Bahasa Indonesia versions: Breastfeeding Knowledge Questionnaire developed by Brodribb, Fallon, Jackson, and Hegney (2008) for measuring knowledge about lactation, less knowledge if score 0–7 and good knowledge if score 8–15. The Iowa Infant Feeding Attitude Scale (IIFAS) developed by Mora and Russel was used to measure attitude towards lactation. Negative attitude if the total score was less than 55 and a positive attitude if the total score was more than 55. Breastfeeding Self-Efficacy Scale-Short Form (BSES-SF), developed by Bandura (1997) used to measure breastfeeding self-efficacy. BSES-SF is an excellent instrument for measuring breastfeeding self-efficacy and is considered appropriate for clinical use such as identifying high-risk breastfeeding mothers, assessing breastfeeding and cognition behaviors as individual strategies in building trust, and evaluate the effectiveness of various interventions and guidelines in development programs (Dennis, 2003). The Cronbach’s alpha coefficient value for each instrument is 0.94, 0.83, 0.9 and 0.87 respectively. Low self-efficacy if total score less than 55 and high self-efficacy if the total score is more than 56.

The study was conducted in Bandung, which is a large city functioning as one of the major economic, commercial and industrial cities in Indonesia and which has various problems resulting from urbanisation. High population density has been shown to be one of the causes of low breastfeeding exclusivity [12]. Breast milk provision in the province of West Java is 35.3%, while the target agreed by the 2015 strategic development was 39% [13].

Univariate analysis was used to describe the characteristics of the respondents. These characteristics include age, education, employment, household income, parity, and delivery type, together with knowledge about and attitude towards breastfeeding, and breastfeeding self-efficacy. A bivariate analysis was conducted using a chi-square test, to gain insight into the relationships between the respondents’ characteristics, knowledge, attitudes and breastfeeding self-efficacy. The multivariate analysis used logistic regression testing to disclose the most dominant factors affecting maternal breastfeeding satisfaction.

## Results

The majority of the respondents were 27–35 years old (65.2%) and had a high level of education (81.4%). They were unemployed (93.1%) and had incomes of less than IDR 2,850,000, i.e., below the regional minimum wage level (75%). The majority are multipara or a parent of multiple children (65.2%). The most common type of delivery is normal per vaginal (85.3%) (see Table 1). Table 2 shows that the majority of respondents claimed to have high maternal breastfeeding satisfaction (53.4%) compared to the rest of the respondents with low satisfaction (46.6%). The majority lacked knowledge on breastfeeding (64.2%). More than half of the respondents had a negative attitude towards breastfeeding (52.9%), but a majority had high self-efficacy (55.9%). The information presented in Table 3 shows that household income, delivery type, and breastfeeding self-efficacy is associated to satisfaction in breastfeeding. The results in Table 4 show that the most dominant factor that affects maternal breastfeeding satisfaction is breastfeeding self-efficacy (OR = 16.64 95% CI 7.6–35.9). Household income, education, attitude and delivery type are also factors that influence maternal breastfeeding satisfaction.

**Table 1:** The Characteristics of the Respondents

Characteristics	Number	Percent
Age		
Young adult (17–25 years old)	71	34.8
Adult (26–35 years old)	133	65.2
Education		
Low level of education (Primary, Middle)	38	18.6
High level of education (High School, College)	166	81.4
Employment		
Unemployed	190	93.1
Employed	14	14
Household Income (IDR)		
Below Regional Minimum Wage	153	75
Above Regional Minimum Wage	51	25
Parity		
Primipara	71	34,8
Multipara	133	65.2
Delivery Type		
Major surgery	30	14.7
Normal (*per vaginal)*	174	65.2

**Table 2:** Maternal breastfeeding satisfaction, knowledge, attitude, and breastfeeding self efficacy

Variables	Number	Percent
Maternal breastfeeding satisfaction		
Low	95	46.6
High	109	53.4
Knowledge regarding breastfeeding		
Insufficient	131	64.2
Sufficient	73	35.8
Attitude toward breastfeeding		
Negative	108	52.9
Positive	96	47.1
Breastfeeding Self-efficacy		
Low	90	44.1
High	114	55.9

**Table 3:** The association between the characteristics of the respondents, knowledge, attitude, breastfeeding self-efficacy, and maternal breastfeeding satisfaction

Independent Variables	Content Variables Maternal Breastfeeding Satisfaction			OR (95% CI)		*p* Value
	High		Low			
	n	%	n	%		
Age						
Young Adult	40	56.3	31	43.7	1.83 (1.02–3.28)	0.058
Adult	55	41.4	78	58.6		
Education						
Low	21	55.3	27	44.7	1.65 (0.80–3.38)	0.234
High	74	44.6	92	55.4		
Employment						
Unemployed	91	47.9	99	52.1	2.30 (0.70–7.56)	0.262
Employed	4	28.6	10	71.4		
Household Income						
Below Minimum	81	52.9	72	47.1	2.97 (1.49–5.94)	0.003
Above Minimum	14	27.5	37	72.5		
Parity						
Primipara	33	46.5	38	53.5	0.99 (0.56–1.77)	1.000
Multipara	62	46.6	71	53.4		
Delivery Type						
Major Surgery	6	20	24	80	0.24 (0.09–0.61)	0.003
Normal (per vaginal)	89	51.1	85	48.9		
Knowledge						
High	56	42.7	75	57.3	0.65 (0.36–1.15)	0.187
Low	39	53.4	34	46.6		
Attitude						
Negative	57	52.8	51	47.2	1.71 (0.98–2.98)	0.081
Positive	38	39.6	58	60.4		
Self-efficacy						
High	71	78.9	19	21.1	14.01 (7.1–27.5)	0.001
Low	24	21.1	90	78.9

**Table 4:** The most dominant factors that affect maternal breastfeeding satisfaction

Variables	*p* value	OR 95% CI
Breastfeeding self-efficacy,^a^	0.000	16.64 (7.6–35.9)
Household income^b^	0.001	4.63 (1.8–11.2)
Education^c^	0.037	2.7 (1.1–7.1)
Attitude^d^	0.046	2.13 (1–4.5)
Delivery type^e^	0.002	0.21 (0.05–0.54)

^a^High vs. low

^b^Below minimum standard vs. above minimum standard

^c^Low vs. high

^d^Negative vs. positive

^e^Major surgery vs. normal (per vaginal)

## Discussion

### Maternal breastfeeding satisfaction

The results indicate that the majority of the respondents (53.4%) experience high maternal breastfeeding satisfaction. They breastfeed for 4 to 8 months after birth. The results of this study are in line with the previous cohort study which stated that the determinants factors of maternal breastfeeding satisfaction at 6 months after delivery are based on the duration of breastfeeding. Longer durations are reported to achieve a higher satisfaction [14]. Breastfeeding is a complex social behaviour, and the mother’s perception of the success of breastfeeding cannot be entirely explained based on whether breastfeeding problems arise; mothers do gain satisfaction, but they also face problems arising from breastfeeding [15]. It is anticipated that high satisfaction leads to longer duration of breastfeeding [9] which in turn impacts on experience and practice with subsequent infants [16].

Gregory, Butz, Ghazarian, Gross, and Johnson mentioned that satisfaction associated with expectations when prenatal about the duration of breastfeeding. The study reported one-year postpartum, 34.7% of the 1802 respondents had met prenatal expected breastfeeding duration, and 23.9% were still breastfeeding. 40.4% respondents reported being satisfied with the duration. But at 12 months postpartum more respondents said that their expectation had been fulfilled, and they are satisfied with the duration of breastfeeding. The results of this study indirectly illustrated that some of the respondents have breastfed for more than 6 months and meet the expectations of breastfeeding after childbirth [17].

The results of this research also show that most of the respondents experience low satisfaction in breastfeeding (46.6%). It is possible that the young adult respondents need guidance for breastfeeding and are primipara without prior experience in the breastfeeding. A minority had undergone caesarean sections, and lactation was not promptly initiated. Also, low breastfeeding satisfaction may have been caused by problems which arose from breastfeeding itself. The most common problems are a scarcity of breast milk and mothers having to work [18]. Besides this, mothers with breastfeeding problems tend to be less satisfied with breastfeeding but are more satisfied with their identity and lifestyle [9].

### Factors associated with breastfeeding satisfaction

Based on statistical testing, there is an association between household income and breastfeeding satisfaction. Some respondents earn below the regional minimum wage. There is a possibility that an association between socio-economic status and breastfeeding is complex, and the difference between these aspects can be tied in with low knowledge about breast milk or feeding, negative attitudes, and no prior feeding experience. However, this research does not support this, in that it suggests that there is no association between household income, education and breastfeeding [19].

The reasons of the mothers with low household income to choose to breastfeed are natural and best for the infant, it improves the health of the mother and child, it is easy to do, and it can enhance the mother-child bonding [20]. Nonetheless, this research does not support these claims but rather states that mothers with higher household income or with better-educated partners, and mothers who have a partner and who are employed are more likely to breastfeed than mothers whose partner or themselves are unemployed [21].

The delivery-type showed association with breastfeeding satisfaction. High breastfeeding satisfaction in a normal delivery may occur because the mother can initiate early breastfeeding. Early breastfeeding initiation is the first step in breastfeeding success. This result rebuts those who state that there is no association between caesarean section and breastfeeding completion and duration, as put forward by Shawkey and Abalkhail [22]. Support for this rebuttal is given by Scott et al.’s prospective cohort research [23] on 1,059 Australian women identifying the influential factors in breastfeeding initiation and duration. They found no association between delivery type and breastfeeding, in that mothers in Australia who underwent caesarean section tended to breastfeed longer compared to those who gave normal delivery [23].

Self-efficacy is associated with breastfeeding satisfaction and is one of the crucial factors in breastfeeding [24]. Mothers who are breastfeeding confidently at the beginning of the post-partum stage tend to do it for longer and have higher self-efficacy in exclusive breastfeeding [25]. These results indirectly provide this interpretation since most of the participant who breastfed for more than 6 months had children already. The results are agreed by Febrianan [26], who claims that there is a relationship between the number of children a woman has education level and experience of breastfeeding and self-efficacy. Self-efficacy or self-confidence is a psychological factor identified as a significant predictor of intention, duration, and exclusivity of breastfeeding [25].

### Influencing factors of breastfeeding satisfaction

Self-efficacy is a dominant factor influencing breastfeeding satisfaction. It is possible that the mothers have prior experience and feed for a long duration are aware of the importance regarding physical and psychological benefits of breastfeeding for their infants and themselves. The confidence and the perception of the mother about the infant feelings of satisfaction during feeding support breastfeeding successfully. It is also the most important determining factors in exclusive breastfeeding [27]. Additionally, self-efficacy supports initiation and duration [28].

Household income also affects breastfeeding satisfaction. Most mothers in this study did not work. Non-working mother has more time with their infants and can breastfeed to meet their infants’ demands. Mothers who frequently breastfeed can maintain milk production and gain direct benefits for their infants and themselves. Household income is one of the factors that contribute to maintaining intention [29]. The results of the research are congruent with researchers who state that the mother’s age, education, family type, family income, and employment status are demographic and social factors that influence initiation and duration of breastfeeding [30].

Another influencing factor in breastfeeding satisfaction is education. Education has a positive impact on breastfeeding because mothers are provided with information on breastfeeding and the application of best practice. The mother’s education is considered to be the most significant factor in the practice of exclusive breastfeeding [30]. In line with the study aforementioned, a high level of education is also the most strongly determining factor for initiation of breastfeeding. Meanwhile, higher parity is the most crucial factor in the continuation of breastfeeding [31].

Similarly, attitude affects satisfaction. A negative attitude is possibly linked to the duration of breastfeeding. Some respondents usually breastfeed for 6 months or more and decided to provide formula as a supplement for nutrition. Attitude is a key determinant of behaviour and a positive attitude towards breastfeeding is required to start and maintain its practice [32]. Furthermore, it is an influential factor for successful breastfeeding in the first 3 months after birth [15].

Delivery types also affect breastfeeding satisfaction. The results highlight that the majority of the respondents gave birth naturally (vaginally). Normal delivery provides greater opportunities for breastfeeding and direct mother-infant contact, as well as combined nursing care. Delivery type is independently predicted to have effects on the duration of breastfeeding, and breastfeeding self-efficacy. It also contributed to formula supplementing in hospitals [33].

#### Limitations of the study

This study is a cross-sectional research study in which independent and dependent variables were evaluated simultaneously without a follow-up procedure. It does not describe events experienced by the mothers before the study regarding breastfeeding success or problems that may have affected their breastfeeding satisfaction. Therefore, it does not disclose the causes and effects of high or low maternal breastfeeding satisfaction.

#### Nursing implications

This study emphasizes the importance of maternal breastfeeding satisfaction of the completion of its provision, especially for the self-efficacy of mothers. Breastfeeding self-efficacy is not the only factor that affects maternal breastfeeding satisfaction and the decrease in exclusive breastfeeding in Indonesia. However, mothers who have breastfeeding self-efficacy receive psychological benefits. They are aware that breastfeeding is important and they have a strong commitment to breastfeeding their infants. Expectant mothers with high incomes can be provided with a choice or guidance and motivation to give birth at maternity hospitals and other places that support consistent breastfeeding.

Nurses or other healthcare professionals can provide information about breast milk or breastfeeding suitable for the comprehension or education level of the information recipient, to foster willingness as well as positive attitudes towards breastfeeding. In maternity wards, healthcare professionals should provide differentiated procedures for early breastfeeding initiation for the various types of delivery (caesarean section or per vaginal). As healthcare professionals, nurses have a significant role to play in continuously encouraging exclusive breastfeeding and conveying information to the public about its importance regarding both physical and psychological benefits.

## Conclusion

Breastfeeding satisfaction is the satisfying feeling obtained during breastfeeding resulting from cooperation between the mother and the infant to fulfil desires or needs. It elevates the hormones oxytocin and prolactin, resulting in the formation of the bond between the mother and infant and promoting happiness which is beneficial for mental and emotional health. Satisfaction in breastfeeding mothers cannot stand alone because there are external and internal influencing factors in play. In this research, it was found that these factors consist of breastfeeding self-efficacy (OR = 16.581), household income (OR = 4.6), education (OR = 2.732), attitude (OR = 2.135) and delivery type (OR = 0.174).

Guidance in lactation by healthcare professionals and breastfeeding counsellors during the seven pre- and post-natal contacts must be implemented for the mother to obtain early information about breast milk and feeding. In maternity training, lactation management knowledge and skills should be developed especially regarding psychological benefits for the mother and the infant. One of the psychological benefits of breastfeeding is satisfaction. This condition indirectly motivates the mother to continue breastfeeding for up to two or more years. The author also recommends further study using different designs and methods with the same instruments to obtain a description of the results in achieving the mother’s role, satisfaction in the mother’s lifestyle and the infant’s satisfaction, as components of maternal breastfeeding satisfaction.

## Data Availability

Detailed data will not be shared due to the confidential nature of the data.

## Abbreviations

BSES-SF: Breastfeeding self-efficacy scale-short form
IIFAS: Infant feeding attitude scale
MBFES: Maternal breastfeeding evaluation scale
UNICEF: United Nations Children’s Fund
